# A Randomized Controlled Study of an HMWHA‐Containing Mask in Skin Repair Following Fractional CO_2_ Laser Resurfacing

**DOI:** 10.1111/jocd.71264

**Published:** 2026-11-05

**Authors:** Zongzhou Wu, Yun Wang, Wei Li, Yu Shi, Yijie Zheng, Zhong Shen, Ruiping Wang, Wei Zhang

**Affiliations:** ^1^ Department of Medical Cosmetology, Shanghai Skin Disease Hospital, School of Medicine Tongji University Shanghai China; ^2^ Department of Hematology Shanghai Central Hospital of Putuo District Shanghai China; ^3^ L'Oreal Dermatological Beauty China, La Roche‐Posay Shanghai China; ^4^ Department of Clinical Research Center, Shanghai Skin Disease Hospital, School of Medicine Tongji University Shanghai China

**Keywords:** ablative fractional CO_2_ laser, clinical trial, erythema index, high‐molecular‐weight hyaluronic acid, transepidermal water loss

## Abstract

**Objective:**

The main objective of this study was to evaluate the overall clinical efficacy of a high‐molecular‐weight hyaluronic acid (HMWHA)‐containing mask in improving skin damage in subjects after ablative fractional laser treatment.

**Methods:**

Subjects who underwent ablative fractional CO_2_ laser treatment were randomly assigned to two groups for postoperative repair intervention: the control group used a self‐made simple cream, and the treatment group used a HMWHA‐containing mask plus the single cream. The pre‐laser skin condition was assessed. The primary outcome measures included transepidermal water loss and erythema index of the facial skin in different intervention groups after laser treatment. Secondary outcome measures included melanin index and epidermal hydration of the facial skin in different intervention groups after laser treatment; clinical assessment of postoperative skin status, including erythema, edema, and desquamation; and subjects' product experience, including burning sensation, itching, and pain. The time points for testing the HMWHA‐containing mask on facial skin were 0.5 h after surgery and postoperative Days 1, 3, 7 ± 1, 14 ± 1, and 28 ± 2. Repeated‐measures ANOVA (for skin instrument test data) and the Wilcoxon rank‐sum test (for subjective ordinal rating data) were used for comparative analysis of the assessment data, and LSD posttests following two‐way ANOVA were applied to continuous time‐series facial data. A difference was considered statistically significant at *p* < 0.05.

**Results:**

A total of 105 subjects were enrolled, including 64 females and 41 males; 25 participants were screened out and did not enter the study (17 females and 8 males), and 80 subjects completed the clinical observation. The subjects were aged 21–39 years, with a mean age of 28.10 ± 4.67 years. In both the control group and the treatment group, TEWL (transepidermal water loss) values showed a marked peak on Day 1, then decreased significantly on Days 3 and 7, which was associated with laser‐induced skin barrier damage. In both groups, epidermal moisture measurements showed a distinct downward trend on Days 1 and 3, which was opposite to the trend in TEWL values and was related to the skin barrier repair process after laser treatment. On Days 3, 7, 14, and 28, epidermal hydration in the treatment group was higher than that in the control group, indicating that the intervention enhanced repair of skin barrier. The erythema index (EI) in both groups decreased over time; on Day 3, the treatment group showed a significant reduction compared with the control group, with a statistically significant difference (*p* < 0.05). The melanin index (MI) in both groups increased on Day 3 and then gradually declined on Days 7, 14, and 28.

**Conclusion:**

This trial showed that the HMWHA‐containing mask can alleviate erythema and edema after ablative fractional CO_2_ laser treatment of the face, reduce transepidermal water loss, maintain skin surface hydration, reduce desquamation, shorten recovery time, and promote rapid repair of skin damage caused by fractional CO_2_ laser treatment.

## 1 Introduction

Traditional ablative laser resurfacing improves scars and photoaging. CO_2_ lasers (10 600 nm) and erbium lasers (2940 nm) are widely used in light skin, but their use is limited in populations with higher Fitzpatrick skin types [1]. Since the application of focal photothermolysis to clinical practice [2] this issue has improved. Fractional CO_2_ laser therapy has become a treatment for various types of scars and photoaging. As an ablative technique, fractional CO_2_ laser can create numerous microscopic thermal zones on the skin surface, induce a local inflammatory response, stimulate fibroblast activation, and promote the regeneration and remodeling of collagen and elastic fibers. These mechanisms work together to help soften scars, reduce scar thickness, and improve scar texture and pigmentation [3]. However, ablative fractional lasers still have relatively high rates of adverse effects in people with darker skin tones, such as persistent facial erythema, hyperpigmentation, hypopigmentation, and prolonged downtime for healing [4]. Ablative fractional CO_2_ laser is a laser technique that ablates the epidermis and superficial dermis, and the integrity of the epidermal barrier is affected after treatment [5]. Ablation and thermal injury histologically form a carbonized layer and a zone of thermal damage [2] The presence of a carbonized layer in the skin is more likely to cause persistent hyperpigmentation in people with darker skin tones [6]. Therefore, rapid repair of the postoperative skin barrier is of great significance for reducing postoperative adverse reactions.

Appropriate perioperative management is crucial for laser treatment because it can reduce or prevent adverse reactions and improve therapeutic outcomes. Postoperative care after fractional CO_2_ laser treatment mainly focuses on promoting healing, maintaining moisture, and sun protection, with particular emphasis on restoring skin barrier function. This helps alleviate postoperative skin reactions, prevent skin infections, accelerate tissue repair, and promote wound healing [7]. Therefore, a series of repair products with anti‐inflammatory and moisturizing effects may be beneficial after CO_2_ fractional laser treatment. For wound healing, moist healing provides a basis for post‐ablative fractional laser repair, and its main advantage is keeping the skin wound in a moist microenvironment [8].

Hyaluronic acid (HA) is a non‐sulfated glycosaminoglycan composed of alternating D‐glucuronic acid and N‐acetyl‐D‐glucosamine [9]. It is widely distributed in connective tissue and skin. HA usually exists as a high‐molecular mass in the synovial fluid surrounding joints, cartilage, and eye tissue and has been shown to play an important role in skin repair [10]. As a natural moisturizing component, it can absorb up to 1000 times its own weight in water. Studies have shown that after 6 weeks of use in patients with facial photoaging, HA can increase skin moisture by 134% immediately and maintain a 55% improvement [11]. High‐molecular‐weight hyaluronic acid (HMWHA) is an ideal moisture‐retaining ingredient. In normal tissues, HA exists in the form of high‐molecular‐weight HA, with an average size ranging from 1 to 10 × 10^6^ Da. Due to its hydrophilicity, HMWHA functions as a structural hydrating polymer. HMW‐HA acts as a molecule that indicates tissue integrity and regulates cellular inflammatory responses [12]. For example, HMWHA can protect mice from T cell‐mediated liver injury and bleomycin‐induced lung injury and can promote the suppressive effects of regulatory CD4+ CD25+ T cells [13, 14]. Hyaluronic acid promotes angiogenesis by stimulating endothelial cell activity, ensuring an adequate blood supply to regenerating tissue. Its water‐retention capacity further supports cell proliferation and migration, accelerating wound closure [11]. Wound healing involves a series of finely regulated steps, during which dynamic interactions occur among the wound, blood components, extracellular matrix, and the cells involved in healing. Multiple cytokines and signaling pathways regulate these responses. In the later stages, the healing process consists of a series of smooth, continuous, and overlapping reactions, in which HMWHA exerts anti‐inflammatory effects and promotes tissue repair [15]. Hyaluronic acid has become a key focus in regenerative medicine and dermatology. Its ability to regulate inflammation, promote fibroblast activity, and enhance vascularization makes it a promising therapeutic agent for optimizing skin repair [11, 16]. The hyaluronic acid mask used in this study contains 2 mg/mL high‐molecular‐weight hyaluronic acid.

Evaluate the comprehensive clinical effect of HMWHA‐containing mask on improving skin damage in subjects who have undergone fractional ablative laser surgery. Assess the safety of the mask for the subjects and evaluate their impact on adherence to auxiliary treatment.

## 2 Methods

### 2.1 Study Design and Subjects

Subjects with facial atrophic scars after acne or moderate to severe facial enlarged pores, who are eligible for fractional CO_2_ laser surgery, and who agree to undergo ablative fractional CO_2_ laser treatment; only subjects who have signed the informed consent form and meet the inclusion and exclusion criteria may be considered for enrollment.

*Inclusion criteria*: Fractional CO_2_ laser treatment is available for adults aged 18–40 with acne‐induced atrophic scars or moderate to severe enlarged pores on their face. Participants must have a full understanding of the trial content, be willing to participate, comply with medical advice and attend follow‐up appointments, sign an informed consent form, and demonstrate good treatment compliance, communication skills, and comprehension abilities.

*Exclusion criteria*: Subjects currently participating in another interventional clinical study; those with diseases that severely affect wound healing; individuals with allergies or allergic constitution to the components of this product; pregnant or breastfeeding women; those with a history of significant organ diseases such as heart, brain, lung, liver, or kidney; those with a history of psychological and mental disorders; individuals with infections at the treatment site; those with active herpes simplex.

*Laser treatment*: After enrollment, before treatment, the subjects' faces were evenly coated with compound lidocaine cream and occluded for 30 min. Immediately after cleansing, the treating physician used a fractional CO_2_ laser in DEEP mode, with an energy setting of 55 mJ/cm^2^ and a fractional coverage area of 5% to treat the entire face.

Participants enrolled in the study will use the product and complete follow‐up visits and questionnaires at baseline and on postoperative Days 1, 3, 7 ± 1, 14 ± 1, and 28 ± 2. The primary study endpoint is postoperative Day 28.

The investigational products are HMWHA‐containing mask, and “Simple Cream.”

*Sample size calculation*: This study is a parallel randomized controlled clinical trial with the primary evaluation indices being the erythema index and transepidermal water loss. The sample size was calculated using mean comparison: a two‐sided confidence interval of 95% and power of 90%, with a 1:1 control. Referring to the previous preliminary experimental results, the erythema index score for the control group (participants who only used a single cream after laser surgery) was 264.67, while the treatment group (participants who used a high‐molecular‐weight mucopolysaccharide medical dressing + a **simple** cream) scored 241.33. Using OpenEPI to calculate, the sample size for both the control and treatment groups in this study should be no less than 35 participants each. Considering the potential loss to follow‐up and the need for sample size based on clinical efficacy evaluation, the total sample size was set at 80 participants, with 40 participants in the experimental group, and 40 in the control group.

Eighty participants will be randomly assigned to two groups, the control group and the treatment group, with 40 cases in each group, and allocation concealment will be implemented using the random envelope method. Forty copies each of the control group (Group A) and treatment group (Group B) schemes will be prepared, sequentially numbered, and placed into 80 opaque envelopes. The envelopes will be sequentially numbered, opaque, and sealed. After a participant enters the study, one envelope will be randomly drawn and opened, and the participant will be enrolled and receive the intervention according to the number inside the envelope. To ensure equal sample sizes in the two groups, whenever a participant dropout is confirmed, the sequentially numbered envelopes for that group will be replenished in a timely manner.

### 2.2 Study Design and Intervention Methods

Immediately after receiving carbon dioxide ablative fractional laser treatment, the following products were used: the control group applied the hospital‐prepared “simple cream” to the entire face; the treatment group used a HMWHA‐containing mask for 15 min, then removed it, gently wiped away any excess film fluid with the fingers, and then applied the “simple cream” to the entire face. Before the scabs fall off, use the following products: In the control group, clean the skin morning and evening with **La Roche‐Posay** spray and sterile cotton swabs, then apply the “simple cream” to the entire face, 2 mL each time, along with strict physical sun protection.

In the treatment group, clean the skin morning and evening with **La Roche‐Posay** spray and sterile cotton swabs. Every night, apply the HMWHA‐containing mask for 15 min and then remove it; gently wipe away any excess film solution with your fingers, then apply the “simple cream” to the entire face. Every morning, use “simple cream” alone, 2 mL each time, along with strict physical sun protection. After the scab has fallen off, use the following products: the control group cleaned the face with **La Roche‐Posay** Toleriane Foaming Cleanser every morning and evening, hydrated with a mist spray, and applied the “simple cream” to the entire face, 2 mL each time, along with sunscreen, wearing a hat, and using an umbrella for sun protection.

The treatment group cleaned the face with **La Roche‐Posay** Toleriane Foaming Cleanser every morning and evening, hydrated with a mist spray, used a HMWHA‐containing mask every night for 15 min and then removed it, gently wiped away any excess film liquid with the fingers, and then applied the “simple cream” to the entire face; every morning, they used “simple cream” alone, 2 mL each time, along with **La Roche‐Posay** sunscreen, wearing a hat, and using an umbrella for sun protection.

Assessment of skin repair status included: subjective evaluation at 0.5 h after surgery, and subjective and objective evaluations, as well as instrumental testing, on postoperative Days 1, 3, 7 ± 1, 14 ± 1, and 28 ± 2. For instrumental testing, a transparent film the size of A4 paper was used for positioning based on the eyebrows and tip of the nose, and a facial simulation board was used to symmetrically select three 1 cm‐radius areas on the left and right cheeks for testing. At the end of the trial, the skin repair effects of the two groups were comprehensively compared.

The primary indicators for detection and observation included the erythema index and transepidermal water loss (TEWL) of the subjects' facial skin. The secondary indicators included the melanin index and epidermal moisture content of the subjects' facial skin. Clinical assessment of the subjects' postoperative skin condition included erythema, edema, and desquamation, as well as questionnaire scores for burning, itching, and pain after product use.

### 2.3 Trial Treatment and Data Collection

The sites for participant information collection, fractional CO_2_ laser treatment, participant information evaluation, and testing were at shanghai skin disease hospital. The intervention was conducted in a separate room with a strictly controlled room temperature of 2°C and indoor humidity of 50%–60% for testing, and the room was well lit.

#### 2.3.1 Improvement in Erythema Index (EI) and Melanin After Product Use

At the baseline visit (Day 0), as well as during follow‐up visits on Days 1, 3, 7 ± 1, 14 ± 1, and 28 ± 2 after surgery, skin moisture content was measured. Three fixed facial points were selected using a facial simulation board to measure the hemoglobin and melanin values of these three areas. Measurements were performed using the CK Company Mexameter MX18, which uses narrow‐band reflectance measurement. The measurement is based on the absorbance values of green light (wavelength 568 nm), corresponding to the spectral absorption peak of hemoglobin, and red light (wavelength 660 nm), corresponding to the spectral absorption peak of melanin, thereby independently outputting the erythema index (EI) and melanin index (MI). The measurement range of the EI is typically 0–999: the lower the value, the paler the skin and the milder the inflammatory/blood flow response. The higher the value, the redder the skin, indicating more pronounced capillary dilation, inflammatory response, or congestion. The EI value of healthy skin is usually between 200 and 400. If the value significantly exceeds 500 or increases substantially compared with the baseline value, it usually suggests skin damage or sensitivity. Before measurement, subjects should sit quietly for 10–15 min, and the skin should be in a resting state. For each subject, three sets of data were measured in each measurement area each time, and the average value was recorded.

#### 2.3.2 Instrument Assessment After Product Use–Tewameter‐Transepidermal Water Loss (TEWL)

At the baseline visit (Day 0), and at follow‐up visits on postoperative Days 1, 3, 7 ± 1, 14 ± 1, and 28 ± 2, TEWL was measured. Three fixed facial regions were marked using a facial simulation board, and the TEWL values of these three regions were measured at fixed points. Measurements were taken using a Tewameter from CK. In skin physiological parameter assessment, TEWL is the most important objective indicator for evaluating skin barrier function, and it measures the rate at which water evaporates through the skin into the external environment. This study used the Tewameter TM 300 from CK (Courage+ Khazaka). The instrument uses an open‐chamber indirect measurement method, applying a hollow cylindrical Tewameter probe to calculate the humidity gradient between points at two different heights, thereby determining the amount of water lost per unit time and per unit area. The unit is g/h/m^2^ (grams/hour/square meter). The lower the TEWL value, the more intact the skin barrier; the higher the value, the faster the water loss and the more severe the barrier damage. 0–10: very healthy, with extremely intact barrier function; 10–20: normal/good, ordinary healthy skin; 21–30: barrier‐damaged/irritated sensitive skin, mild skin inflammation; > 30: seen in conditions with severe barrier damage such as atopic dermatitis, psoriasis, and laser injury. In this study, after the subject had been seated quietly for 10 min, the operator should wear a mask and measurements must be taken in a windless environment to avoid the values undergoing a period of intense fluctuation after the probe contacts the skin. It is necessary to wait until the curve stabilizes, after which the software automatically takes the average of the stabilized values. For each subject, three sets of data were collected for each measurement area at each measurement, and the average value was recorded.

#### 2.3.3 Instrumental Assessment After Product Use (Corneometer—Skin Moisture Content)

At the baseline visit (Day 0), as well as at follow‐up visits on Days 1, 3, 7 ± 1, 14 ± 1, and 28 ± 2 after surgery, skin moisture content was measured. A facial simulation plate was used to fix three predefined facial sites, and the TEWL values of these three areas were measured. Measurements were performed using the Corneometer from CK, specifically the Corneometer CM 825 from the German company CK, which uses a capacitance method based on the large difference in dielectric constants between water and other substances. The instrument display typically ranges from 0 to 120 (an arbitrary unit, not a percentage): < 30: Very Dry; 30–45: Dry; > 45: Sufficiently Hydrated. During measurement in this study, the probe was held perpendicular to the skin with constant pressure until the spring compression reached its limit. A rapid 1‐s measurement was performed, and the probe was lifted immediately after the reading was locked to avoid prolonged pressure causing a local “occlusion effect” that could increase skin moisture. After each measurement, the metal probe surface had to be cleaned with a dry tissue. For each subject, three measurements were taken at each measurement site per session, and the mean value was recorded.

#### 2.3.4 Investigator Clinical Assessment—Erythema, Edema, and Desquamation

At 0.5 h after ablative fractional CO_2_ laser treatment, and at follow‐up visits on Days 1, 3, 7 ± 1, 14 ± 1, and 28 ± 2 postoperatively, the clinical trial physician used the Erythema Visual Analog Scale (Erythema VAS) to assess skin erythema: no skin erythema = 0 points, mild skin erythema = 1 point, moderate skin erythema = 2 points, and severe skin erythema = 3 points; used the Erythema Visual Analog Scale (Erythema VAS) to assess skin edema: no skin edema = 0 points, mild skin edema = 1 point, moderate skin edema = 2 points, and severe skin edema = 3 points; and used the Erythema Visual Analog Desquamation (Erythema VAS) to assess skin desquamation: no skin desquamation = 0 points, mild skin desquamation = 1 point, moderate skin desquamation = 2 points, and severe skin desquamation = 3 points.

#### 2.3.5 Subjective Sensation Assessment—Burning, Itching, Pain

At 0.5 h after ablative fractional CO_2_ laser treatment, and at follow‐up visits on Days 1, 3, 7 ± 1, 14 ± 1, and 28 ± 2 after treatment, subjects completed an assessment of posttreatment facial sensations, including burning, itching, and pain. Each sensation was rated using a 0–10 visual analog scale. At the 28‐day follow‐up, the subjects completed a questionnaire regarding compactness, thickness, breathability, comfort, odor, stickiness, hygroscopicity, humidity, smoothness, and overall evaluation.

#### 2.3.6 Visia Photography

At the baseline visit (Day 0), 0.5 h after the ablative fractional CO_2_ laser procedure, and during follow‐up visits on postoperative Days 1, 3, 7 ± 1, 14 ± 1, and 28 ± 2, photographs were taken using Visia (Canfield, USA). For each subject and each measurement area, three sets of data were collected at each time point, and the average value was recorded.

#### 2.3.7 Statistical Methods

Demographic data, baseline participant data, and treatment compliance data will be analyzed descriptively. Normally distributed data will be described as mean ± SD, while non‐normally distributed data will be described using the median and interquartile range. For skin instrument measurement data on postoperative Days 1, 3, 7 ± 1, 14 ± 1, and 28 ± 2, repeated‐measures ANOVA will be used. Comparative analysis of subjective rating data will be performed using the Wilcoxon rank‐sum test. For continuously measured facial data over time, the LSD test in two‐way ANOVA will be applied. A difference will be considered statistically significant when *p* < 0.05.

## 3 Results

A total of 105 participants were recruited and screened, including 64 females and 41 males. Among them, 25 participants were excluded and did not enter the study (17 females and 8 males), including 8 participants who did not meet eligibility criteria, 10 who did not meet diagnostic criteria, 3 who were not between 18 and 40 years old, and 4 who declined to participate (as shown in Figure 1). 80 participants completed the ablative fractional CO_2_ laser surgery and completed clinical observation. The participants' ages ranged from 21 to 39 years, with an average age of 28.10 ± 4.67 years. The treatment group consisted of 40 participants, with an average age of 28.5 ± 4.85, including 27 participants under 30 years old (67.5%), 13 participants over 30 years old (32.5%), 16 participants males (40%), and 24 participants females (60%). The control group consisted of 40 participants, with an average age of 26.9 ± 3.04, including 34 participants under 30 years old (85%), 6 participants over 30 years old (15%), 19 participants males (47.5%), and 21 participants females (52.5%). There was no statistically significant difference in age and gender distribution between the two groups.

**FIGURE 1:**
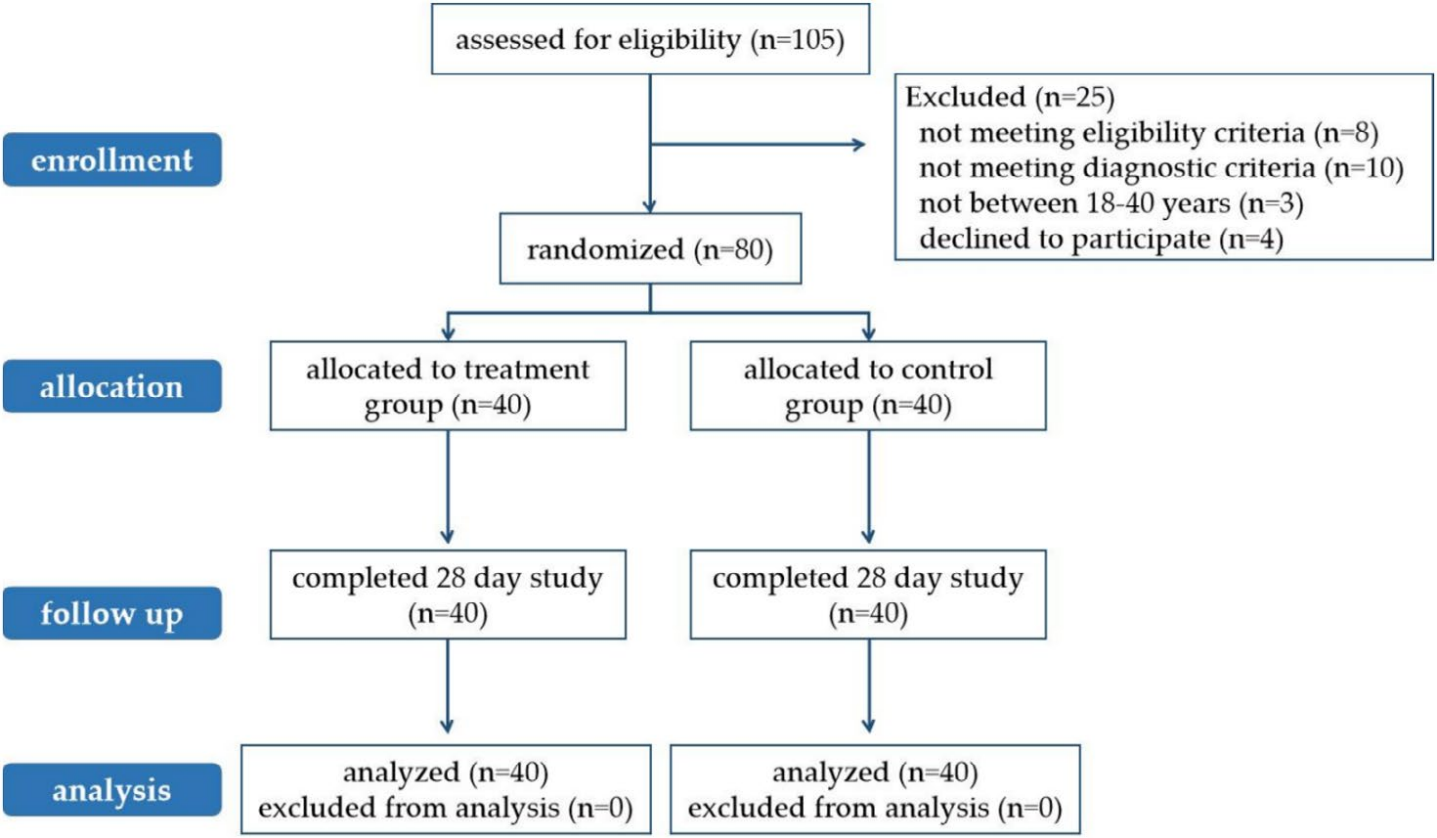
Clinical trial process.

Evaluation index at baseline: The treatment group's erythema index mean ± SD = 294.1 ± 49.2, while the control group's erythema index was 310.1 ± 46.4. The treatment group's TEWL mean ± SD = 12.9 ± 4.9, while the control group's TEWL mean ± SD = 13.1 ± 3.4. There was no statistically significant difference between the two groups in the main detection indices at baseline (T0). The treatment group's melanin index (MI, T0), mean ± SD = 126.7 ± 32.5, while the control group's melanin index (MI, T0), mean ± SD = 127.4 ± 34.6. The treatment group's skin moisture content (T0) mean ± SD = 55.4 ± 10.4, while the control group's skin moisture content (T0) mean ± SD = 52.4 ± 10.8. There was no statistically significant difference between the treatment and control groups in MI and skin moisture content at baseline (T0). The treatment group's edema score (T0), median 1.0, IQR 1.0–2.0; the control group's edema score (T0), median 2.0, IQR 2.0–3.0, *χ* ^2^ = 5.790, *p* = 0.000. There was a statistically significant difference between the two groups, but it was a non‐primary observation index (as shown in Table 1).

**TABLE 1:** Demographic features of patients with fractional CO_2_ laser treatment.

Variables	Treatment group (*n* = 40)	Control group (*n* = 40)	*t*/*z*/*χ* ^2^	*p*
Age (years), (mean, SD)	28.5 (4.85)	26.9 (3.04)	1.713	0.091
Age (years), (median, IQR)	27.5 (25.5–32.0)	26.5 (25.0–29.0)	1.324	0.186
Age (years), *n* (%)			3.382	0.066
< 30	27 (67.5)	34 (85.0)		
≥ 30	13 (32.5)	6 (15.0)		
Sex, *n* (%)			0.457	0.499
Male	16 (40.0)	19 (47.5)		
Female	24 (60.0)	21 (52.5)		
Evaluation index at baseline				
Erythema index (E, T0), (mean, SD)	294.1 (49.2)	310.1 (46.4)	1.497	0.139
Melanin index (MI, T0), (mean, SD)	126.7 (32.5)	127.4 (34.6)	0.102	0.919
TEWL (T0), (mean, SD)	12.9 (4.9)	13.1 (3.4)	0.167	0.868
Skin moisture content (T0), (mean, SD)	55.4 (10.4)	52.4 (10.8)	1.267	0.209
Edema (T0), (median, IQR)	1.0 (1.0–2.0)	2.0 (2.0–3.0)	**5.790**	**0.000**
Desquamation (T0), (median, IQR)	0 (0–0)	0 (0–0)	1.000	0.317

*Note:* T0: Time for the baseline evaluation instantly implemented after the fractional CO_2_ laser treatment. The *p* value is less than or equal to 0.05, indicating that there is a statistically significant difference between the two groups.

Abbreviations: IQR, interquartile range; SD, standard deviation; TEWL, transepidermal water loss.

In both the control group and the treatment group, TEWL values showed a significant peak on Day 1, followed by a significant decrease on Days 3 and 7, which was related to laser‐induced skin barrier damage. In both groups, epidermal water measurement exhibited a noticeable downward trend on Days 1 and 3, which was contrary to the trend of TEWL values and was associated with the skin barrier repair process after laser treatment (shown in Figure 2). On Days 3, 7, 14, and 28, the treatment group had higher epidermal hydration than the control group, indicating that the intervention enhanced the skin barrier repair (skin appearance shown in Figure 3). The EI in both groups decreased over time; on Day 3, the treatment group showed a significant reduction compared to the control group, with a statistically significant difference (*p* < 0.05). The MI in both groups increased on Day 3 and then gradually decreased on Days 7, 14, and 28 (in Table 2).

**FIGURE 2:**
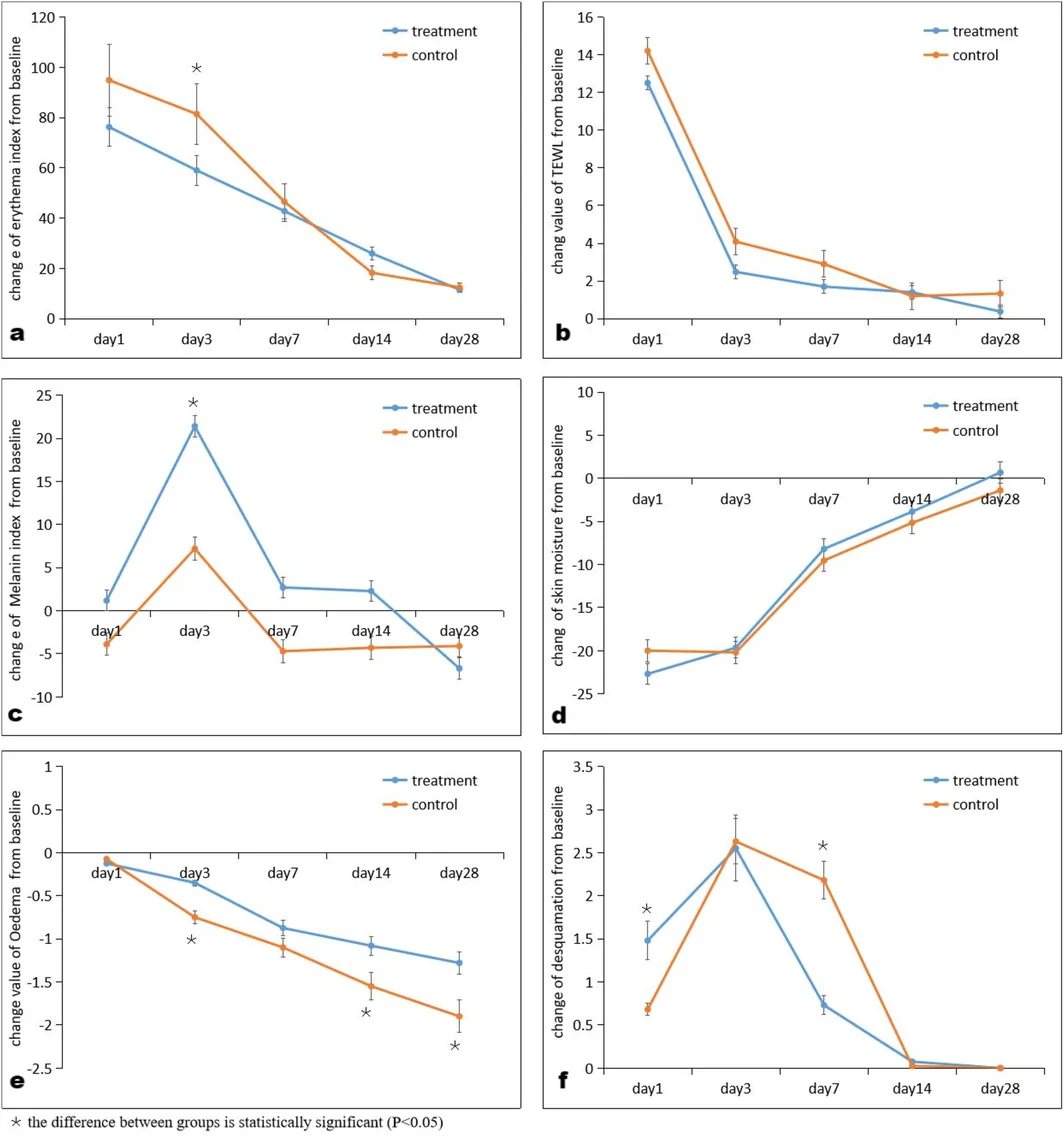
In the control group, single cream was used, and in the test group, single cream + mask was used twice daily. Different assessment and measurement data were collected after the intervention at postoperative Day 1 (D1), Day 3 (D3), Day 7 (D7), and Day 28 (D28): (a) changes in erythema index, (b) changes in TEWL values, (c) changes in melanin index, (d) changes in skin moisture content, (e) changes in skin edema scores, and (f) changes in skin desquamation.

**FIGURE 3:**
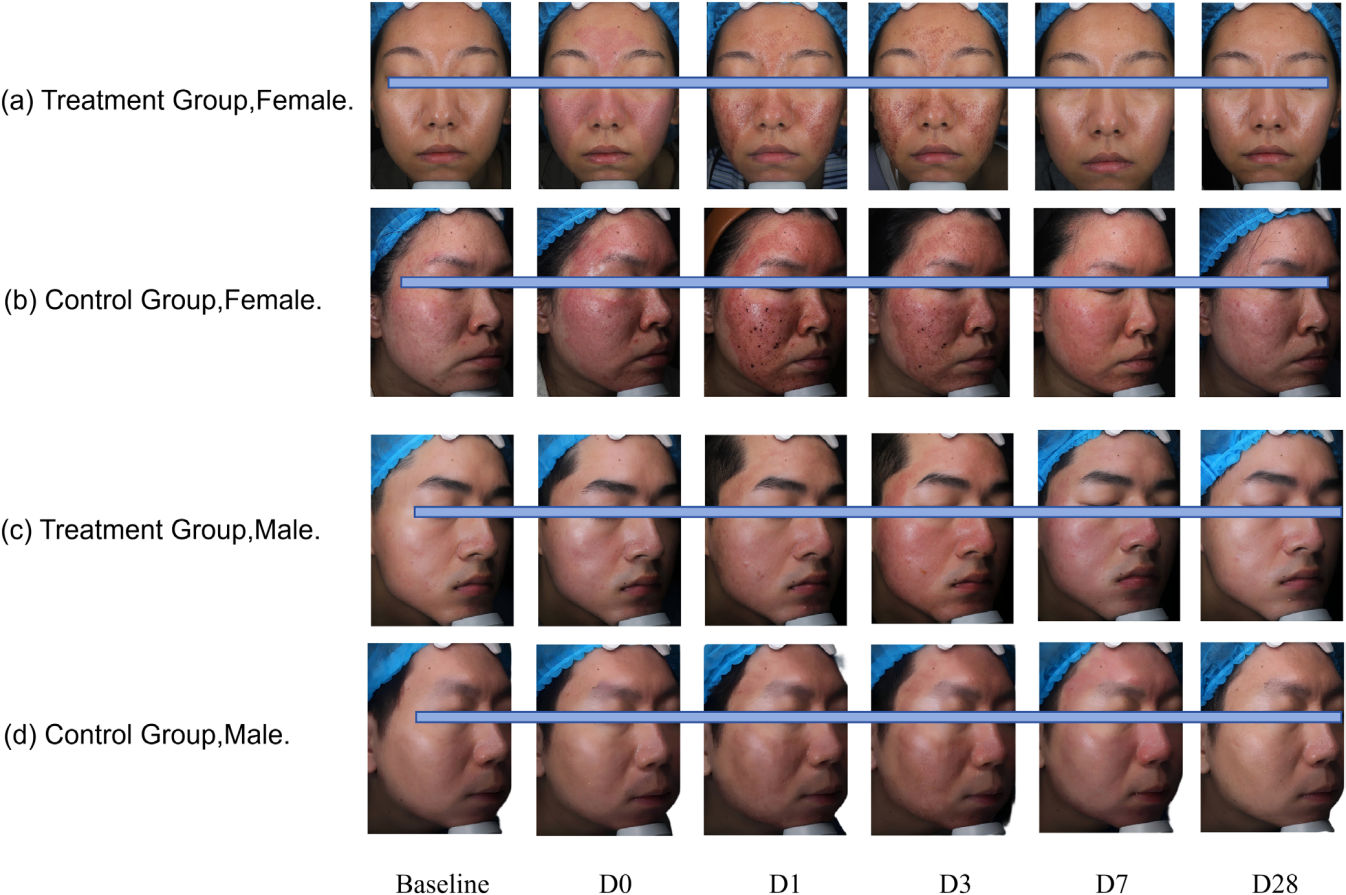
Representative VISIA photographs illustrating the repair process of facial skin after fractional CO_2_laser treatment. Photographs were obtained before treatment (baseline, D0) and at postoperative day 1 (D1), day 3 (D3), day 7 (D7), and day 28 (D28). Panels: (a) Treatment Group, Female; (b) Control Group, Female; (c) Treatment Group, Male; (d) Control Group, Male.

**TABLE 2:** The intervention effect of mask among patients with fractional CO_2_ laser treatment.

Variables	Treatment group (*n* = 40)	Control group (*n* = 40)	Difference (95% CI)	*p*
**Primary outcome**				
Erythema index at Day 3, mean (SD)	353.1 (51.8)	391.5 (58.9)	−38.4 (−63.1 to −13.7)	0.003
Erythema index at Day 3, median (IQR)	356.3 (318.5–378.3)	375.3 (348.3–442.0)	NA	0.007
Change at Day 3, mean (SD)	58.9 (45.7)	81.4 (54.4)	−22.4 (−44.8 to −0.1)	0.047
**Secondary outcomes**				
Erythema index at Day 1, mean (SD)	370.3 (49.4)	404.9 (60.9)	−34.7 (−59.4 to −9.9)	0.007
Erythema index at Day 7, mean (SD)	336.9 (48.9)	356.6 (41.2)	−19.8 (−39.9 to 0.3)	0.054
Erythema index at Day 14, mean (SD)	320.0 (54.9)	328.3 (34.8)	−8.3 (−28.8 to 12.2)	0.398
Erythema index at Day 28, mean (SD)	305.6 (37.9)	322.5 (57.2)	−16.9 (−38.5 to 4.7)	0.123
Melanin index at Day 1, mean (SD)	127.8 (42.7)	123.5 (38.2)	4.3 (−13.7 to 22.4)	0.634
Melanin index at Day 3, mean (SD)	148.0 (44.7)	134.7 (37.8)	13.4 (−5.1 to 31.8)	0.153
Melanin index at Day 7, mean (SD)	129.3 (54.8)	122.7 (34.8)	6.7 (−13.7 to 27.1)	0.517
Melanin index at Day 14, mean (SD)	128.9 (38.9)	123.1 (34.7)	5.8 (−10.6 to 22.3)	0.481
Melanin index at Day 28, mean (SD)	119.9 (35.5)	123.3 (37.2)	−3.3 (−19.5 to 12.8)	0.682
TEWL at Day 1, mean (SD)	25.5 (5.8)	27.3 (6.1)	−1.8 (−4.5 to 0.8)	0.177
TEWL at Day 3, mean (SD)	15.4 (4.9)	17.2 (5.8)	−1.8 (−4.2 to 0.6)	0.145
TEWL at Day 7, mean (SD)	14.6 (4.9)	15.9 (5.3)	−1.3 (−3.5 to 0.9)	0.252
TEWL at Day 14, mean (SD)	14.3 (4.9)	14.3 (4.7)	0.1 (−2.1 to 2.2)	0.956
TEWL at Day 28, mean (SD)	13.3 (4.4)	14.4 (3.9)	−1.1 (−3.0 to 0.8)	0.240
Skin moisture content at Day 1, mean (SD)	32.7 (7.5)	32.4 (9.1)	0.3 (−3.4 to 4.0)	0.864
Skin moisture content at Day 3, mean (SD)	35.7 (9.1)	32.1 (8.3)	3.6 (−0.3 to 7.5)	0.069
Skin moisture content at Day 7, mean (SD)	47.2 (10.6)	42.8 (14.6)	4.4 (−1.3 to 10.0)	0.131
Skin moisture content at Day 14, mean (SD)	51.5 (10.4)	47.2 (12.3)	4.3 (−0.8 to 9.4)	0.096
Skin moisture content at Day 28, mean (SD)	56.1 (10.5)	50.9 (9.8)	5.1 (0.6 to 9.6)	0.027
Edema at Day 1, median (IQR)	1.0 (1.0–1.0)	2.0 (2.0–3.0)	NA	0.000
Edema at Day 3, median (IQR)	1.0 (1.0–1.0)	1.0 (1.0–2.0)	NA	0.000
Edema at Day 7, median (IQR)	0 (0–0)	1.0 (1.0–1.0)	NA	0.000
Edema at Day 14, median (IQR)	0 (0–0)	1.0 (0.0–1.0)	NA	0.000
Edema at Day 28, median (IQR)	0 (0–0)	0 (0–1.0)	NA	0.001
Desquamation at Day 1, median (IQR)	1.5 (0.0–3.0)	1.0 (0.0–1.0)	NA	0.003
Desquamation at Day 3, median (IQR)	3.0 (2.0–3.0)	3.0 (3.0–3.0)	NA	0.666
Desquamation at Day 7, median (IQR)	0.5 (0.0–1.0)	3.0 (1.0–3.0)	NA	0.000
Desquamation at Day 14, median (IQR)	0 (0–0)	0 (0–0)	NA	0.549
Desquamation at Day 28, median (IQR)	0 (0–0)	0 (0–0)	NA	1.000

Abbreviations: CI, confidence interval; IQR, interquartile range; NA, not applicable; SD, standard deviation; TEWL, transepidermal water loss.

The survey results on mask usage among 40 subjects in the treatment group indicated that there was no statistically significant difference (*p* > 0.05) in the evaluation of the mask's compactness, thickness, breathability, comfort, smell, absorbance, moisture, smoothness, and overall evaluation between 16 male subjects and 24 female subjects (in Table 3). However, the evaluation of the sticky sensation showed a lower satisfaction among males, with a statistically significant difference between genders (*p* < 0.05).

**TABLE 3:** Experience and satisfaction for mask use among patients with fractional CO_2_ laser treatment.

Indicators	Total participants in treatment group (*n* = 40)	Male participants (*n* = 16)	Female participants (*n* = 24)	*p*
Compactness [median, (IQR)]	4.0 (3.0–5.0)	4.0 (3.5–4.0)	4.0 (3.0–5.0)	0.967
Thickness [median, (IQR)]	4.0 (4.0–5.0)	4.0 (4.0–5.0)	4.0 (3.5–5.0)	0.652
Breathability [median, (IQR)]	4.0 (3.5–5.0)	4.0 (4.0–5.0)	4.0 (3.0–5.0)	0.436
Comfortableness [median, (IQR)]	5.0 (4.0–5.0)	5.0 (4.0–5.0)	5.0 (4.0–5.0)	0.967
Smells [median, (IQR)]	5.0 (4.0–5.0)	5.0 (4.0–5.0)	5.0 (4.0–5.0)	0.946
Sticky sensation [median, (IQR)]	4.0 (4.0–5.0)	3.0 (3.0–4.5)	4.0 (4.0–5.0)	**0.036**
Absorbance [median, (IQR)]	4.0 (4.0–5.0)	4.0 (4.0–5.0)	4.5 (4.0–5.0)	0.672
Moisture[median, (IQR)]	4.5 (4.0–5.0)	4.0 (4.0–5.0)	5.0 (4.0–5.0)	0.420
Smoothness [median, (IQR)]	4.0 (4.0–5.0)	4.0 (4.0–5.0)	4.5 (4.0–5.0)	0.795
Overall evaluation [median, (IQR)]	4.0 (4.0–5.0)	4.0 (4.0–5.0)	4.0 (4.0–5.0)	0.774

*Note:* The *p* value is less than or equal to 0.05, indicating that there is a statistically significant difference between the two groups.

Abbreviation: IQR, interquartile range.

## 4 Discussion and Conclusion

In this study, subjects with post‐acne scars and enlarged pores of Fitzpatrick skin types III–V underwent fractional CO_2_ laser treatment. The skin‐repair effects after intervention with HMWHA‐containing mask were observed. The results showed that TEWL values in both the control and treatment groups peaked significantly on Day 1 of use and then decreased on Days 3 and 7, which was associated with laser‐induced skin barrier damage. The epidermal moisture measurements in both groups showed a marked downward trend on Days 1 and 3, opposite to the trend in TEWL values, indicating that the skin barrier was in the process of repair after laser treatment. On Days 3, 7, 14, and 28, the treatment group had higher epidermal water content than the control group, suggesting that the experimental intervention enhanced the recovery of skin barrier function. The EI in both the control and treatment groups decreased over time; on Day 3, the treatment group was significantly lower than the control group, with a statistically significant difference (*p* < 0.05). The MI in both groups increased on Day 3 and then gradually declined on Days 7, 14, and 28; on Day 3, the treatment group differed significantly from the control group (*p* < 0.05), showing an interaction effect. Previous studies have reported that the incidence of post‐inflammatory hyperpigmentation (PIH) after fractional CO_2_ laser treatment varies widely, ranging from 0% to 100%, and it is generally believed that patients with higher Fitzpatrick skin types are at greater risk [17, 18] This increased susceptibility is mainly attributed to the higher melanin content in darker skin, which makes individuals more prone to melanocyte hyperactivity and post‐inflammatory pigmentary changes [19].

Compared with other moisturizers such as polyethylene glycol, glycerol, ethylene glycol, and sorbitol, the advantage of the HMWHA material used in this study is that it is not affected by relative humidity and exhibits excellent water‐retention performance under both high‐ and low‐humidity conditions [20]. Since researchers confirmed in a porcine model that a moist environment can accelerate wound healing, an increasing number of clinicians have begun to adopt moist wound healing for skin wound care [21]. A moist environment can prompt wounds to initiate the healing process more quickly [22], enabling keratinocytes to rapidly migrate to the wound surface and complete epithelial regeneration. In a dry environment; however, they migrate to the subcutaneous tissue beneath the scab. When the skin is injured, cells communicate with one another by secreting growth factors and other signaling molecules to repair tissue in an orderly manner. These molecules require a liquid medium for intercellular communication, thereby promoting healing [23]. A moist environment can retain the tissue proteases contained in wound exudate, thereby promoting the dissolution and absorption of necrotic tissue and relieving pain [24]. In addition, it can reduce inflammation at the wound site and lower the risk of scar formation [25]. A moist environment does not increase the risk of wound infection because it can effectively isolate the wound from the external environment. Some studies have shown that wounds treated with occlusive moist masks have lower bacterial counts than those treated with dry masks. By contrast, dry scabs contain large numbers of biofilm bacteria [26]. As a barrier against external contaminants, masks effectively protect wounds from microbial invasion and reduce the risk of secondary infection, playing a crucial role in wound care [27].

Erythema is a common postoperative reaction after laser treatment. HA plays a key role in regulating angiogenesis and is an effective modulator of endothelial cell (EC) function. Native HA, at physiological concentrations, can inhibit endothelial cell proliferation and disrupt cell–cell/cell‐matrix interactions. Angiogenic oligosaccharides can promote endothelial cell proliferation and migration, possibly through receptor‐mediated mechanisms [28]. The mask used in the study contains HMW‐HA, which can also enhance Foxp3 expression in Tregs and promote anti‐inflammatory cytokines such as IL‐2, IL‐10, and TGF‐β [29]. A clinical trial demonstrated that 0.2% sodium hyaluronate cream is effective in treating rosacea and improving erythema, tolerability, and compliance [30]. The results showed that the erythema index in the HA group improved significantly at all three follow‐up time points, suggesting that HA plays a role in regulating angiogenesis, inflammation, and antioxidant activity. In addition to its moisturizing and anti‐inflammatory properties, HA also exhibits significant analgesic effects. HA modulates transient receptor potential vanilloid 1 (TRPV1) channels, reducing nociceptor activity and pain signaling [31].

Our recent study used masks containing hyaluronic acid of different molecular weights. Our recent research utilized different molecular HA masks. A total of 60 patients were included. On Day 14, the TEWL in the HA group was significantly lower than in the control group and the percentage change in TEWL during the period of the most significant reduction was also greater in the HA group than in the control group. The EI in the HA group was significantly lower than those in the control group on 3, 7, and 14 days. The study highlights HA with varied molecular sizes enhances post‐laser recovery by reducing TEWL and erythema, alleviating pain, and promoting healing [32]. The results of a split‐face controlled study published by Berardesca et al. [33] in 2020 on the benefits of hyaluronic acid masks for patients with rosacea showed that masks containing hyaluronic acid could improve skin moisture content, reduce transepidermal water loss, thereby repairing the skin barrier, and at the same time alleviate skin erythema, a symptom of redness and inflammation. Zhang M used a hyaluronic acid‐based hydrogel with excellent self‐healing ability and enhanced mechanical properties; this hydrogel had a fast gelation rate, controllable swelling behavior, good biocompatibility, and enhanced mechanical strength similar to that of human dermis, and also achieved significant repair effects [34].

Ablative fractional CO_2_ laser treatment is commonly chosen to evaluate the efficacy and safety of certain repair products, because ablative CO_2_ fractional laser treatment creates wounds with exudation, edema, and even bleeding. However, compared with ablative CO_2_ laser treatment, the wound is smaller and there is no obvious risk of infection, so it is relatively widely used in people with darker skin tones. Post‐laser repair after ablative CO_2_ fractional laser treatment is the best choice for evaluating the safety of a repair product and its effect on moist wound healing of micro‐wounds. Since persistent erythema, transepidermal water loss, and surface moisture content during post‐laser repair are related to the subject's age, scar area, pore size, and laser energy settings [35] in this study the subjects' ages were limited to the range of 18–40 years, and the laser parameters were standardized and unified.

This study has certain limitations. First, to promote wound healing after CO_2_ laser surgery, both study groups used a simple moisturizing cream. Although this approach is clinically reasonable for the management of open wounds, it may itself have facilitated the timely restoration of skin barrier function, thereby markedly reducing the data differences between the two groups. The lack of a moist masks control may introduce potential confounding effects. This study was not designed as a split‐face study. Although we used covariance analysis to adjust for potential confounding factors, individual differences could not be completely excluded as a source of interference with the results. Second, using more objective and quantifiable assessment methods, such as dermoscopy, VISIA imaging, and optical coherence tomography (OCT), would help improve the reliability of the results. Third, the follow‐up period was limited, and extending the follow‐up would help further evaluate the effect of hyaluronic acid on pigmentation. Finally, there has been no comprehensive evaluation of histopathological and molecular studies that reveal the main mechanisms of action of this skincare product.

## 5 Conclusion

In conclusion, this prospective randomized controlled trial demonstrates that the mask used in this trial caused no adverse reactions or SAE events throughout the study period. Compared with the control group, it offered advantages in reducing transepidermal water loss, maintaining skin surface moisture, and shortening the time to recovery from skin desquamation. It also reduced erythema after CO_2_ fractional laser treatment and alleviated postoperative discomfort, suggesting that its combined use with laser therapy is beneficial for promoting postoperative skin recovery. For patients after laser treatment, this product is easy to use, safe, and reliable, and readily suitable for clinical application.

## Author Contributions

**Zongzhou Wu:** conceptualization, methodology, data curation. **Yun Wang:** data curation, writing – original draft preparation. **Wei Li:** writing – review and editing, funding acquisition. **Yu Shi:** writing – review and editing, funding acquisition. **Yijie Zheng:** writing – review and editing, funding acquisition. **Zhong Shen:** writing – review and editing, funding acquisition. **Wei Zhang:** project administration, supervision. **Ruiping Wang:** statistical analysis.

## Disclosure

All patient data and images have been thoroughly anonymized and blinded to ensure that the patient cannot be identified.

## Ethics Statement

Reviewed and approved by the Ethics Committee of Shanghai Skin Disease Hospital (No. 2021–30 (scientific)). The patients in this manuscript have given written informed consent to the publication of their case details.

## Consent

Written informed consent was obtained from the patient(s) for the publication of this case report and any accompanying images.

## Conflicts of Interest

The authors declare no conflicts of interest.

## Data Availability

The data that support the findings of this study are available from the corresponding author upon reasonable request.
